# Corneal Higher-Order Aberrations after Microhook ab Interno Trabeculotomy and Goniotomy with the Kahook Dual Blade: Preliminary Early 3-Month Results

**DOI:** 10.3390/jcm10184115

**Published:** 2021-09-12

**Authors:** Hiromitsu Onoe, Kazuyuki Hirooka, Hideaki Okumichi, Yumiko Murakami, Yoshiaki Kiuchi

**Affiliations:** Department of Ophthalmology and Visual Science, Hiroshima University, 1-2-3 Kasumi, Minami-Ku, Hiroshima 734-8551, Japan; onoehir@hiroshima-u.ac.jp (H.O.); okumic@hiroshima-u.ac.jp (H.O.); ymiko00@hiroshima-u.ac.jp (Y.M.); ykiuchi@hiroshima-u.ac.jp (Y.K.)

**Keywords:** higher-order aberrations, ab interno trabeculotomy, Kahook Dual Blade, glaucoma

## Abstract

We examined postoperative corneal higher-order aberrations (HOAs) present after combined phacoemulsification with either microhook ab interno trabeculotomy (μLOT-Phaco) or goniotomy, using the Kahook Dual Blade (KDB-Phaco). Retrospective study: A total of 45 eyes underwent μLOT-Phaco and KDB-Phaco (LOT-Phaco) procedures, with 21 eyes that underwent cataract surgery alone used as controls. Visual acuity and corneal HOAs, coma-like aberrations, and spherical-like aberrations were analyzed before and at 1, 2, and 3 months after the surgeries. Risk factors that could potentially influence HOAs were evaluated. No significant postoperative changes were noted for corneal HOAs, coma-like aberrations, and spherical-like aberrations after cataract surgery alone. The mean corneal HOAs, coma-like aberrations, and spherical-like aberrations were 0.222 ± 0.115 μm, 0.203 ± 0.113 μm, and 0.084 ± 0.043 μm at baseline and 0.326 ± 0.195 μm (*p* < 0.001), 0.302 ± 0.289 μm (*p* = 0.03), and 0.150 ± 0.115 μm (*p* < 0.001) at 3 months after LOT-Phaco, respectively. Results of the analysis for risk factors suggested that a longer incision in Schlemm’s canal could influence corneal HOAs, coma-like aberrations, and spherical-like aberrations after LOT-Phaco. Although no significant postoperative changes were observed in corneal HOAs and coma-like or spherical-like aberrations after cataract surgery alone, a significant increase in corneal HOAs and coma-like or spherical-like aberrations remained after the LOT-Phaco procedure.

## 1. Introduction

Glaucoma is the leading the cause of blindness [1]. Although medication and laser therapy alone are used to try to decrease the intraocular pressure (IOP), incisional surgery is performed when these prove to be ineffective. The trabecular meshwork has been shown to be the most resistant part of the aqueous humor outflow, with glaucomatous eyes found to have a greater resistance in this area [2]. Theoretically, when attempting to improve the control of the IOP, incision or removal of the trabecular meshwork should result in a reduction in this resistance [3]. Thus, the use of devices, such as the trabectome, Kahook Dual Blade (KDB; New World Medical, Rancho, Cucamonga, CA, USA), and microhook (Inami & Co., Ltd., Tokyo, Japan), in conjunction with 5-0 nylon sutures has been utilized during attempts designed to reduce the resistance of the trabecular meshwork from within the anterior chamber [4,5,6,7]. These techniques all fall within the designation of minimally invasive glaucoma surgery (MIGS) and can be performed by using procedures that utilize the iridocorneal angle from within the anterior chamber.

As compared to other vision threatening complications, corneal topographic changes are often less serious. However, the frequently reported vision complaints made by patients could potentially be related to corneal topographic changes. Due to recent advances in wavefront analysis, it has become possible to more closely evaluate ocular surgeries, such as refractive surgery and cataract surgery. Results have shown that there are changes in the higher-order aberrations (HOAs), which were found to be related to the postoperative visual function [8,9]. Moreover, the effect of trabeculectomy on HOAs in glaucoma patients has also been reported [10,11,12]. Increases in the HOAs are believed in general to lead to decreases in the functional visual acuity. However, the specific effect associated with MIGS combined with cataract surgery on ocular HOAs has yet to be definitively established.

The purpose of our current study was to examine patients undergoing phacoemulsification cataract extraction in conjunction with the placement of an intraocular lens (IOL) combined with either μLOT (μLOT-Phaco) or goniotomy with KDB (KDB-Phaco) and then investigate the improvement of the HOAs following surgery.

## 2. Materials and Methods

### 2.1. Patients

Between December 2019 and September 2020, this retrospective study examined eyes undergoing either μLOT-Phaco in 31 eyes or KDB-Phaco in 14 eyes at Hiroshima University Hospital, Japan. Twenty-one eyes undergoing phacoemulsification cataract extraction in conjunction with the placement of an IOL (Phaco) were additionally examined and used as the controls. All patients were followed for at least 3 months after surgery. The Institutional Review Board of the Hiroshima University approved the study protocol (IRB No. E-2240). In accordance with the principles outlined in the Declaration of Helsinki, all subjects provided written informed consent in addition to the standard consent for surgery prior to their enrollment and participation in the research study.

Patients who had previously undergone intraocular surgery were excluded. Inclusion criteria required patients to be ≥50 years of age and have no history of refractive surgery or other significant ocular diseases.

### 2.2. Surgical Techniques

Trabeculotomy was performed by using 2 different kinds of trabecular hooks, the microhook or the KDB. Selection of the hooks used in the surgeries was based on the preferences and decisions of the surgeons. The glaucoma procedure was performed under direct gonioscopy after the cataract procedure in both the μLOT-Phaco and KDB-Phaco patient groups. A 2.8 mm corneal incision was created at the temporal position. A standard phacoemulsification technique; Whitestar Signature Pro (Johnson & Johnson, New Brunswick, NJ, USA) was used to remove the nucleus during all procedures. The IOLs (PCB00V, Johnson & Johnson; XY1, HOYA, Tokyo, Japan) were implanted in the capsular bag. After cataract surgery, a microhook was inserted into the anterior chamber. The inner wall of the Schlemm’s canal and trabecular meshwork were incised by a microhook at 4 clock hours (nasal quadrant: 120°) or at 6 clock hours (inferior and nasal bisection: 180°). The extent of the incision in the Schlemm’s canal in degrees (EIS) was based on decisions of the surgeons. The KDB was introduced into the anterior chamber for the purpose of performing excisional goniotomy, with the pointed chip engaging the trabecular meshwork to the point where its heel was resting within Schlemm’s canal. In order to excise a strip of the trabecular meshwork, the KDB was then advanced approximately 4 clock hours (nasal quadrant: 120°). After removal of the ophthalmic viscosurgical device, a 27-gauge cannula was used to hydrate the incisions. There were no sutures placed or used at the end of the surgery. Postoperative anti-inflammatory and anti-infective therapies were administered for 3 to 4 weeks in all patients.

### 2.3. Wavefront Analysis

A wavefront analyzer (KR-1W, Topcon Co., Tokyo, Japan) was utilized to measure the anterior, posterior, and total corneal wavefront aberrations preoperatively and at 1, 2, and 3 months postoperatively. Aberrometry measurements were performed automatically for a total of three times. Displayed wavefront aberrations included corneal HOAs, trefoil, coma, spherical, third-order, and fourth-order aberrations of the Zernike polynomials, which were calculated as root mean square values. 

### 2.4. Statistical Analysis

A paired *t*-test or chi-square test was used to compare the values for the LOT-Phaco and Phaco alone groups. A multivariable regression model was used to determine the factors that were associated with the increases in the HOAs at 3 months after LOT-Phaco. The independent variables used included the age, EIS (120° or 180°), baseline corneal HOAs, IOP at one day after surgery, IOP at 3 months after surgery, and the device used (KDB or microhook). To ensure that clinically relevant and confounding variables remained, the analysis used backward elimination selection. All data are reported as the mean ± standard deviation (SD). All statistical analyses were conducted by using JMP software version 15 (SAS Inc., Cary, NC, USA). *P*-values less than 0.05 were considered statistically significant.

## 3. Results

Phacoemulsification cataract extraction was performed in 21 eyes, with combined phacoemulsification with trabeculotomy performed in 31 eyes when using the microhook and in 14 eyes when using the KDB. Table 1 presents the clinical characteristics for the enrolled participants. The mean ages for the μLOT-Phaco and KDB-Phaco (LOT-Phaco) groups and the cataract surgery (Phaco) control group were 73.3 ± 8.8 years and 78.9 ± 6.0 years, respectively.

**Figure 1:**
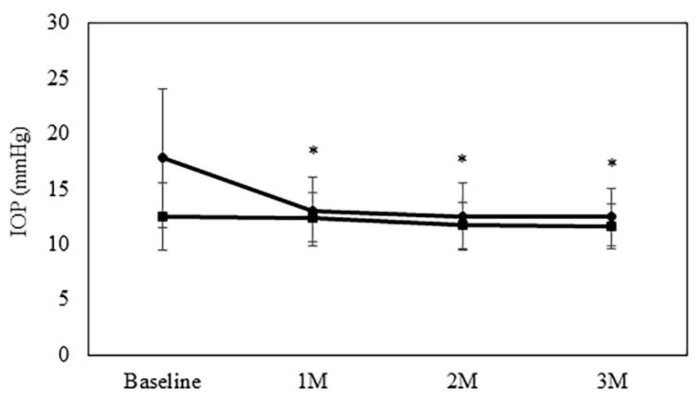
Mean intraocular pressure following Phaco-alone or LOT-Phaco. The intraocular pressure was significantly reduced in LOT-Phaco group compared with baseline. * *p* < 0.05 compared with baseline. Filled circle LOT-Phaco group; filled square Phaco group.

Figure 1 presents the IOP trends noted between the two groups over the study course. The mean IOP in the LOT-Phaco group was 18.0 ± 6.3 mmHg at baseline, while it was 12.9 ± 3.0, 12.3 ± 2.9, 12.6 ± 2.5 mmHg at 1, 2, and 3 months, respectively. The mean IOP in the Phaco group was 11.7 ± 2.5 mmHg at baseline, while it was 12.2 ± 2.6, 11.4 ± 2.4, 11.7 ± 2.1 mmHg at 1, 2, and 3 months, respectively. At all of the study visits, there was a significant reduction noted in the IOP as compared to baseline in the LOT-Phaco group. Table 2 shows the pre- and postoperative visual acuities in both groups. At all study visits in both groups, there was a significant increase observed in the visual acuity as compared to baseline.

**Figure 2:**
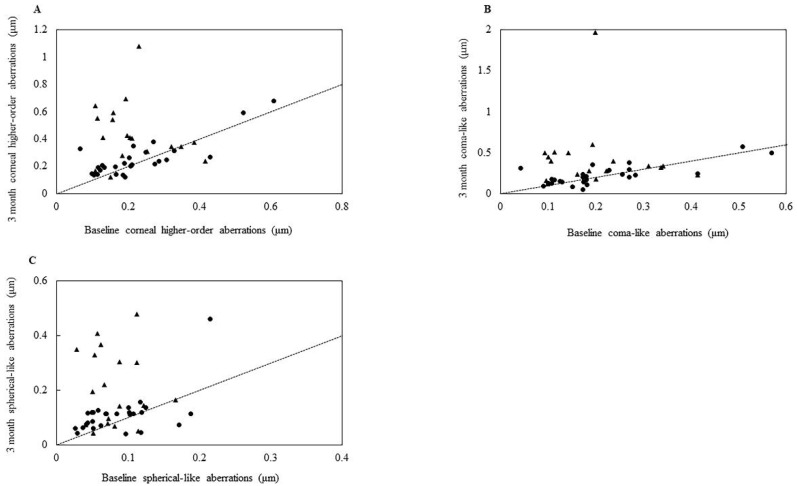
Scatterplot showing baseline versus 3-month corneal higher-order (**A**), coma-like (**B**), or spherical-like aberrations (**C**). Filled circle 120° EIS group; filled triangle 180° EIS group.

Table 3 shows the pre- and postoperative corneal HOAs, coma-like aberrations, and spherical-like aberrations. No significant postoperative changes were observed in the Phaco group for the corneal HOAs and coma-like or spherical-like aberrations. However, corneal HOAs and coma-like or spherical-like aberrations were significantly increased in the LOT-Phaco group at every visit following the initial surgery. Figure 2 demonstrates baseline versus 3 months after LOT-Phaco corneal HOAs (A) and coma-like (B) or spherical-like aberrations (C).

The risk factor that was identified as being associated with the increased corneal HOAs, coma-like aberrations, and spherical-like aberrations at 3 months after LOT-Phaco was EIS for the 180° Schlemm’s canal opening (Table 4).

## 4. Discussion

Recently, procedures using new microsurgical devices are being developed for MIGS [5,7]. MIGS is commonly defined as a surgical procedure that uses an ab interno approach, results in minimal trauma with very little, minimal or no conjunctival manipulation, and has a good safety profile and rapid recovery [13]. However, at 3 months after the LOT-Phaco procedure in our current study, we found that the there was a continued significant increase in the total HOAs and coma-like or spherical-like aberrations.

Changes in the HOAs following trabeculectomy have been reported in several studies [10,11,12]. Fukuoka et al. [10] found that there was an increase in the ocular coma-like and total higher-aberrations incidence at 1 month after trabeculectomy, with these changes shown to return to baseline levels by 3 months. In contrast, we previously reported finding corneal HOAs were significantly increased from baseline up until 3 months after trabeculectomy [11]. Moreover, we also found that hypotony could influence corneal HOAs after filtration surgery [11]. However, in our current study, we found that hypotony was not observed in all cases.

In this current study, eyes undergoing Phaco were used as the controls. Although we did not find any significant postoperative changes in the corneal HOAs and coma-like or spherical-like aberrations after undergoing Phaco, results did show that these parameters were significantly increased following LOT-Phaco. However, the only difference noted between these surgeries was for the incision in Schlemm’s canal. In addition, the 180° more extensive incision in Schlemm’s canal was found to be a risk factor that influenced the corneal HOAs, coma-like aberrations, and spherical-like aberrations. In the 120° Schlemm’s canal incision group, corneal HOAs, coma-like aberrations, and spherical-like aberrations were 0.227 ± 0.128 μm, 0.206 ± 0.124 μm, and 0.086 ± 0.050 μm at baseline and 0.251 ± 0.131 μm (*p* = 0.12), 0.217 ± 0.123 μm (*p* = 0.47), and 0.110 ± 0.077 μm (*p* = 0.10) at 3 months after LOT-Phaco, respectively. When compared to the baseline values, there were no significant differences observed. In the 180° Schlemm’s canal incision group, however, corneal HOAs, coma-like aberrations, and spherical-like aberrations were 0.216 ± 0.095 μm, 0.198 ± 0.098 μm, and 0.080 ± 0.034 μm at baseline and 0.439 ± 0.223 μm (*p* = 0.001), 0.430 ± 0.406 μm (*p* = 0.04), and 0.211 ± 0.136 μm (*p* = 0.002) at 3 months after LOT-Phaco, respectively. Based on these findings, we assume that Schlemm’s canal plays an important role in maintaining corneal formation.

Corneal HOAs and coma-like or spherical-like aberrations were significantly higher than baseline until 2 months (data not shown) and were no longer significantly different from the baseline at 3 months in the 120° Schlemm’s canal incision group. Therefore, we assume that those aberrations in the 180° Schlemm’s canal incision group may also return to baseline if we extend the follow-up period.

Current study limitations included, first, the effect of the corneal thickness, axial length, type of implanted IOL, and eccentricity in each eye. However, it is our belief that the influence of these factors is probably low. Second, it is known that age has an influence on corneal HOAs [14]. The mean age in our current study was significantly different between the LOT-Phaco and Phaco groups. However, we found no influence of age for HOAs following the surgery in our current study. Third, although increases in HOAs are believed to lead to decreases in functional visual acuity, our current study did not evaluate the functional visual acuity, with only the visual acuity evaluated. However, we are presently undertaking an evaluation of the vision-related quality of life that is present after undergoing the LOT-Phaco procedure. Fourth, the relationship between the EIS and the reduction in IOP remains unclear. The mean IOP reduction in our current study was 21.2 ± 4.8% and 27.3 ± 5.8% (*p* = 0.42) at 3 months after surgery in the 120° EIS and 180° EIS groups, respectively. Manabe et al. [15] previously reported that there was no correlation between the EIS (range 150° to 320°) during suture trabeculotomy and the postoperative reduction in the IOP. Thus, a further long-term comparison of the postoperative outcomes of EIS in a larger number of patient cases will need to be undertaken in the future.

In conclusion, even though the corneal HOAs and coma-like or spherical-like aberrations exhibited no significant increase from the baseline in the Phaco group, the corneal HOAs and coma-like or spherical-like aberrations in the LOT-Phaco group remained significantly increased from the baseline for up to 3 months after the procedure. Thus, a more extensive incision in the Schlemm canal should be considered to be a risk factor that can influence corneal HOAs and coma-like or spherical-like aberrations.

## Figures and Tables

**Table 1:** Baseline patient characteristics.

	LOT-Phaco (*n* = 45)	Phaco (*n* = 21)	*p*-Value
Age (years)	73.3 ± 8.8	78.9 ± 6.0	0.001
Gender (M/F)	16/29	9/12	0.57
Visual acuity (logMAR)	0.28 ± 0.27	0.16 ± 0.19	0.09
Baseline IOP (mmHg)	17.8 ± 6.1	12.5 ± 3.1	<0.001
Diagnosis			
POAG	15		
PACG	19		
Exfoliation glaucoma	8		
Secondary glaucoma	3		
Device (microhook/KDB)	31/14		
EIS (120°/180°)	27/18		

M, male; F, female; IOP, intraocular pressure; POAG, primary open-angle glaucoma; PACG, primary angle closure glaucoma; KDB, Kahook Dual Blade; EIS, extent of the incision in the Schlemm canal in degrees.

**Table 2:** Postoperative logMAR visual acuity.

	LOT-Phaco	*p*-Value	Phaco	*p*-Value
Baseline	0.28 ± 0.27		0.16 ± 0.19	
1M	0.10 ± 0.28	<0.001	0.01 ± 0.13	0.004
2M	0.08 ± 0.27	<0.001	−0.003 ± 0.13	0.001
3M	0.06 ± 0.23	<0.001	−0.03 ± 0.14	<0.001

**Table 3:** Postoperative corneal higher-order, coma-like, and spherical-like aberrations.

	LOT-Phaco	*p*-Value	Phaco	*p*-Value
Corneal higher-order				
Baseline	0.222 ± 0.115		0.230 ± 0.229	
1M	0.297 ± 0.161	<0.001	0.252 ± 0.134	0.44
2M	0.356 ± 0.255	0.002	0.268 ± 0.142	0.21
3M	0.326 ± 0.195	<0.001	0.272 ± 0.137	0.12
Coma-like				
Baseline	0.203 ± 0.113		0.206 ± 0.107	
1M	0.251 ± 0.137	0.004	0.228 ± 0.133	0.44
2M	0.305 ± 0.201	0.003	0.239 ± 0.145	0.26
3M	0.302 ± 0.289	0.03	0.244 ± 0.127	0.14
Spherical-like				
Baseline	0.084 ± 0.043		0.092 ± 0.058	
1M	0.151 ± 0.100	<0.001	0.098 ± 0.050	0.69
2M	0.162 ± 0.174	0.01	0.107 ± 0.050	0.27
3M	0.150 ± 0.115	<0.001	0.113 ± 0.071	0.20

Data shown are mean ± SD (μm).

**Table 4:** Stepwise multiple regression analysis for factors associated with postoperative corneal higher-order, coma-like, and spherical-like aberrations’ changes.

	Corneal Higher-Order				Coma-like				Spherical-like			
	Univariate		Multivariate		Univariate		Multivariate		Univariate		Multivariate	
Factors	β	*p*-Value	β	*p*-Value	β	*p*-Value	β	*p*-Value	β	*p*-Value	β	*p*-Value
Age	−0.003	0.38			−0.004	0.93			−0.002	0.23		
IOP one day after surgery	0.002	0.54			0.006	0.27			0.001	0.63		
IOP 3 months after surgery	0.009	0.45			0.012	0.49			0.005	0.43	0.013	0.04
Device (KDB)	−0.134	0.03			−0.138	0.14			−0.080	0.02		
Baseline corneal HOAs	−0.558	0.03	−0.526	0.02								
Baseline coma-like					−0.570	0.14						
aberrations												
Baseline spherical-like									−0.473	0.23		
aberrations												
EIS (120°)	−0.200	<0.001	−0.098	<0.001	−0.222	<0.001	−0.111	0.01	−0.108	<0.001	−0.064	<0.001

IOP, intraocular pressure; KDB, Kahook Dual Blade; HOAs, higher-order aberrations; EIS, extent of the incision in the Schlemm’s canal in degrees.

## Data Availability

The data analyzed in this study are available from the corresponding author upon reasonable request.
